# Recovery after Running an “Everesting” Mountain Ultramarathon

**DOI:** 10.3390/life13101946

**Published:** 2023-09-22

**Authors:** Anton Ušaj, Jon Lihteneger Vidmajer, Sonja Lojen

**Affiliations:** 1 Laboratory of Biodynamics, Faculty of Sport, University of Ljubljana, 1000 Ljubljana, Slovenia; jon.lihteneger@gmail.com; 2 Department of Environmental Sciences, Institute Jožef Stefan, Jamova 39, 1000 Ljubljana, Slovenia; sonja.lojen@ijs.si

**Keywords:** relative running intensity, energetic cost, lactate threshold, carbohydrate oxidation rate, respiratory fatigue

## Abstract

Blood markers of muscle microdamage and systemic inflammation do not adequately explain the reduced performance observed over a prolonged recovery after running a mountain ultramarathon. This case study aimed to determine whether the reduced performance after the Everesting mountain ultramarathon can be further assessed by considering cardiorespiratory and metabolic alterations determined via repeated incremental and continuous running tests. A single runner (age: 24 years, BM: 70 kg, BMI: 22, Vo_2peak_: 74 mL∙min^−1^∙kg^−1^) was observed over a preparatory period of two months with a one-month recovery period. The Everesting consisted of nine ascents and descents of 9349 vertical metres completed in 18:22 (h:min). During the first phase of the recovery, enhanced peak creatine kinase (800%) and C-reactive protein (44%) levels explained the decreased performance. In contrast, decreased performance during the second, longer phase was associated with a decreased lactate threshold and Vo_2_ (21% and 17%, respectively), as well as an increased energetic cost of running (15%) and higher endogenous carbohydrate oxidation rates (87%), lactate concentrations (170%) and respiratory muscle fatigue sensations that remained elevated for up to one month. These alterations may represent characteristics that can explain the second phase of the recovery process after Everesting.

## 1. Introduction

Everesting ultramarathon running events evolved from the general category of mountain ultramarathons (MUM) and ultramarathons. This running event consists of several uphill and downhill runs that reach or even exceed the cumulative terrestrial altitude of Mount Everest (8864 m). The altitude, the steepness, the nature of the routes (dry land, ice and rocks) and thus a high proportion of eccentric work and different, even extreme environmental conditions are the main differences compared to other ultramarathons. The decreased performance during these runs is a result of accumulated fatigue. Ultramarathons last several hours to days and are interrupted by rest breaks that are as short as possible. Decreased performance has been associated with an increased energy cost (Cr) during this event [1,2], muscle microdamage due to mechanical stress [3,4], oxidative stress [5,6], neuromuscular fatigue [7,8], systemic inflammation [9,10], respiratory muscle fatigue [11] and an imbalance between the effects of the anabolic hormone testosterone and the catabolic hormone cortisol [12]. The changes during an ultramarathon persist after these events in the recovery phase but eventually revert to their resting values. This phenomenon could be related to the return of an athlete’s ultra-endurance performance to pre-competition levels. The most dramatic changes occur in the first few days after an ultra-endurance competition. The typical blood markers for muscle microdamage, represented by creatine phosphokinase (CPK), and for systemic inflammation, represented by C-reactive protein (CRP), have been associated with a significant decrease in performance. Notably, the aforementioned changes reach peak blood concentration about 1–2 days after the ultramarathon and then decrease toward the resting values within the next 3–5 days [13].

In contrast, performance tests used to assess recovery after ultramarathons showed a sustained decline in performance even after a longer period. Sherman et al. [14] reported decreased isokinetic torque during knee extension after as many as seven days. Similarly, Chambers et al. [15] found that vertical jump height decreased for 18 days. Warhol et al. [4] reported that muscle biopsies showed incomplete regeneration 12 weeks after an ultramarathon. Although these parameters and the corresponding performance tests do not represent extreme endurance performance, they suggest that a return to pre-competition performance may take longer than ten days [4,13,16].

The aforementioned dramatic decrease in performance could be associated with an early dramatic increase in the blood markers of muscle damage and systemic inflammation. Despite these phenomena disappearing within days, the performance remained reduced. Hypothetically, the recuperation of performance during the recovery period after an ultramarathon could represent two phases reflecting the typical behaviour of a dynamic system after the termination of an impulse [17]. We hypothesised that the same two phases may also occur after Everesting. The first phase could be related to the early dramatic decrease in performance. The second phase follows the first phase and could be a longer process associated with slow recovery from microdamage in exercising muscles [4]. These phenomena cannot be detected by blood markers measured during resting because they already reach their resting values typical for a period before the ultramarathon, with the exception of the values obtained via muscle biopsy [4]. Therefore, they may be associated with other factors. One of these could be the decrease in running efficiency, as may be evidenced by increased energetic costs. Other markers should also be identified and selected to potentially assess changes in cardiorespiratory function through an increase in heart rate and ventilation and changes in metabolism through an altered selection between carbohydrates and fats as fuels. However, these alterations require adequate exercise, perhaps in an ultramarathon, where they manifest themselves in an organism that has not yet fully recovered. Therefore, we hypothesise that fluctuations in the potential parameters to be selected and tested in advance will be greater after Everesting than during the preparatory period and will disappear in conjunction with the increased performance during the second recovery phase, lasting one month or longer. Testing this hypothesis requires an incremental testing protocol and a continuous running test instead of resting measurements. A continuous running test needs initial, runner-specific calibration according to intensity and duration to ascertain possible differences influenced by training and those caused by Everesting and recovery. These tests should be repeated several times during the experimental procedure before Everesting (preparatory period) and after Everesting (recovery period). Managing a complex experiment needs careful planning, which can enable standardization of testing conditions, but also needs to be flexible to disable interferences with training in the preparatory period and with recovery time course during the recovery period. To achieve these standards effectively, a case study research design was selected.

## 2. Case Report

### 2.1. Participant and Ethical Approval

A single male subject was used in this study, an amateur ultramarathon runner (age: 24 years; BM: 70 kg; BMI: 22; Vo_2peak_ = 74 (ml∙min^−1^∙kg^−1^)). The runner had five years of experience in endurance running. In the last two years, the subject specialised in Everesting. Preparation for the competition season started in November, but special preparation for Everesting started only two months before the event. At the time of the experiment, the runner was healthy and had no injuries. The subject gave his written informed consent, and the experimental procedure was approved by the Faculty of Sport Ethics Committee (FS8:2021).

### 2.2. Experimental Design and Protocol

The preparatory period for the Everesting trial consisted of two months of regular training. The additional month after Everesting represented a recovery period. The runner participated in the following tests: resting haematological venous blood measurements, a body composition test, an incremental test and a submaximal continuous running test (CRT), all on the treadmill. These tests were repeated according to the schedule shown in Table 1.

**Figure 1:**
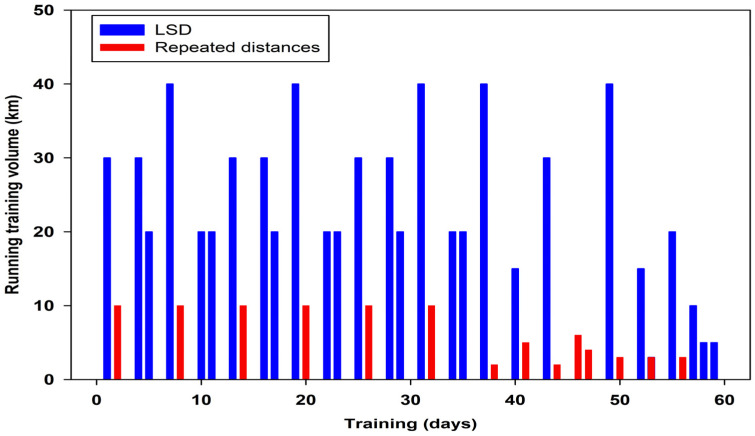
Training characteristics during the preparatory period for Evereting consisted of LSD—long, slow distance and repeated distances.

Training in the last two months before Everesting consisted of two one-month mesocycles. The training characteristics were designed by the runner. The first of the two mesocycles consisted of five 6-day microcycles of equal length. The first day consisted of a continuous 30–40 km run at 3.5–3.8 m∙s^−1^ (LSD) (Figure 1). The fourth day consisted of a similar but shorter continuous 20–30 km run at about 4.6–4.8 m∙s^−1^ (LSD) (Figure 1). The second day of the microcycle consisted of 2 km runs repeated five times at a speed of about 5.1–5.3 m∙s^−1^ (close to Vo_2peak_ intensity), with five minutes of recovery in between (repeated distances) (Figure 1). The third and sixth days were recovery days.

The first day consisted additionally of light stretching and general strength exercises. The fifth training day consisted of about 20 km of fartlek and Nordic walking in the mountains. The fartlek training started with a steady run of light to moderate intensity, followed by two repetitions of about one kilometre of high–intensity running on slightly sloping terrain, and continued with a two–kilometre run with very steep descents. The first half of the training ended with about three-kilometres of very steep Nordic walking. The second half of the training starts with a fast run of four-kilometres downhill, followed by two repetitions of runs of one kilometre uphill. The fartlek ends with a four–kilometre steep descent to the starting place. The first two microcycles of the second mesocycle were a continuation of the microcycles in the first mesocycle. The four other microcycles represented tapering in which the running volume was linearly reduced to the last 60% of the training volume in the first mesocycle (Figure 1). The total training volume in both mesocycles was 750 km.

### 2.3. Measurements

#### 2.3.1. Haematological Measurements

Haematological tests were performed in the morning (fasted). Two venous blood samples of 5 and 3.5 mL were collected in SST II Advance BD Vacutainers and centrifuged at 1700 RPM for 10 min. Analyses were performed using Beckmann Coulter AU 680 (Beckmann, Boston, MA, USA) and Abbott Architect i1000 (Abbott, Abbott Park, IL, USA) devices. The third sample of 3 mL was collected in a BD Vacutainer K2E (Becton Dickinson, Franklin Lakes, NJ, USA) with EDTA. The haemogram was obtained using a Sysmex XN-1000 analyser. The tests were repeated six times according to a specific scheme (Table 1).

#### 2.3.2. Body Composition

Body composition was analysed using an InBody720 Body Composition Analyser (Seoul, Republic of Korea) in the morning (fasted). The test was repeated nine times according to a specific scheme (Table 1).

#### 2.3.3. Incremental Testing Protocol

The incremental test was repeated five times according to a specific schedule (Table 1). This test consisted of 4-min runs on a Pulsar treadmill (HP Cosmos, Nußdorf, Germany). The running speed was increased by 2 km∙h^−1^ from an initial 2.2 m∙s^−1^ (8 km∙h^−1^). This exercise was interrupted for 0.5 min for blood sampling until the subject could no longer maintain his running speed due to fatigue. Continued breath-by-breath gas exchange was analysed using a Vmax Spectra (SensorMedics, Orange City, FL, USA). At every exercise stage, the blood lactate concentration [LA] was measured from a hyperaemic earlobe capillary blood sample (5 μL) using the Lactate Pro 2 analyser (Arkray, Kyoto, Japan).

#### 2.3.4. Continuous Running Test (CRT)

**Figure 2:**
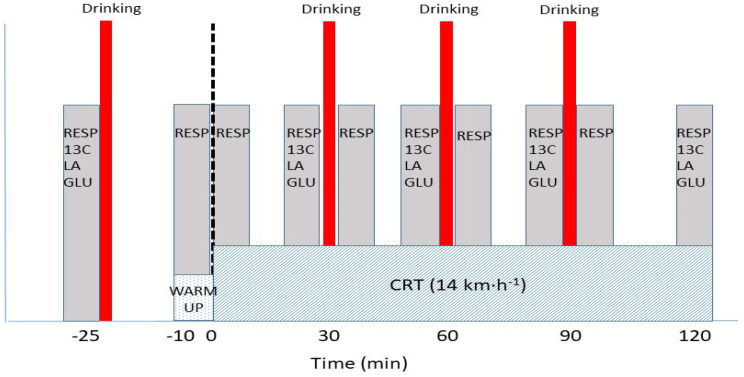
Test setup during CRT. Testing consisted of intervals for collecting breath and isotope samples and for collecting blood micro samples, represented by grey rectangles, and drinking breaks, represented by thick red lines interrupting the continuous run. Testing activities began about 25 min before the start of the CRT with measurements of gas exchange at rest, blood samples and bolus drinking. About 10 min before the test, the second measurements of respiration and gas exchange were taken, followed by a warm-up run. The CRT started at 0 min and continued with interruptions for measurements and drinking until 120 min.

A continuous 120-min submaximal running test (CRT) at 3.9 m∙s^−1^ (14 km∙h^−1^) was performed eight times: four times with a carbohydrate drink (CHO, Xc) and four times with water (WAT, Xw), according to a specific schedule (Table 1). The velocity was selected to correspond with the Lactate Threshold [18,19] determined during the first test, which was classified as “somewhat heavy” according to the Borg scale [20]. A 25-min rest period before the test was followed by a 10-min warm-up run of 2.2 m∙s^−1^ (8 km∙h^−1^), followed by a two-hour run briefly interrupted for measurement purposes (Figure 2). The runner arrived at the laboratory having consumed a diet of foods naturally low in ^13^C for three days. The runner also avoided strenuous endurance training during this time. About 25 min before the start, Capsolin cream (Laboratorio Farmaceutico SIT, Mede, Italy) was applied to the skin of the earlobe to influence hyperaemia. Respiratory gases were also collected at rest in a comfortable sitting position using a Vmax Spectre metabolic cart (Sensor Medics, Yorba Linda, CA, USA). In addition, two vacutainers (20 mL each) were filled with exhaled air to determine the ^13^C/^12^C ratio of exhaled air at rest. A micro-sample of 95 µL capillary blood from a hyperaemic earlobe was collected to measure blood gas, acid–base, electrolyte, lactate [LA], and glucose [GLU] concentrations using an ABL800FLEX analyser (Radiometer, København, Denmark). The runner ingested a 6 mL∙kg^−1^ bolus of a 15% sugarcane solution (CHO) (Mascavo, Brusque, Brazil) 20 min before the start of the test. The sugarcane consisted of 87% sucrose, 2% glucose and 2% fructose with a high natural abundance of ^13^C (*δ*^13^C_VPDB_ = −9.8 ‰). Breath analysis to determine the ^13^C/^12^C ratio was performed using a Europa Scientific 20–20 isotope ratio mass spectrometer with an ANCA- TG trace gas separation module (Europa Scientific, Cheshire, UK). The test run began with a 10-min warm-up at 2.8 m∙s^−1^ (Figure 2), which continued at 3.9 m∙s^−1^ (14 km∙h^−1^) until the first interruption for approximately 20–30 s at 25 min to remove the breathing mask and put on a mask adapted for isotope breath sampling. The runner continued to run until the 27th minute. Breath sampling was performed in the last three minutes. Then, the run was interrupted for about 1 min to collect blood samples, consume the drink (this time, 2 mL∙kg^−1^) and put the breathing mask back on (Figure 2). The run continued until the second interruption, this time between the 45th and 50th minutes (and again in the interval between the 55th and 60th minutes) to remove and put on the breathing mask to collect breath for the isotope measurement (Figure 2). The third repetition of the procedure occurred between the 75th and 80th minutes and again in the interval between the 85th and 90th minutes. The fourth and final repetition was performed between the 105th and 110th minutes and again in the interval between the 115th and 120th minutes (Figure 2). The last blood samples were taken immediately after the end of the run.

#### 2.3.5. Calculations

CHO and FAT oxidation rates were calculated using the standard indirect calorimetry equations [21,22]. From the measured Vo_2_ and Vco_2_, the total CHO and FAT oxidation rates were calculated as follows:

CHOox = 4.55 · Vco_2_ − 3.21 · Vo_2_

FATox = 1.67 · Vo_2_ − 1.67 · Vco_2_.

We applied the assumption that protein oxidation during exercise was negligible.

The isotope composition is expressed as the ‰ difference between the ^13^C^/12^C of the sample and a known laboratory reference standard [23,24] according to the following:

δ=((C13/C12)sample(C13/C12)standard −1)·103 [‰]

where δ is the relative deviation of the heavy-to-light C isotope ratio of the sample from that of the standard (VPDB), expressed in per mil (‰). The EXOox (g∙min^−1^) was then calculated [25] as

$$EXOox=\frac{V_{CO2}\;\cdot \;(\delta exp-\delta b)}{(\delta ing-\delta b)\;}\;\cdot \;\frac{1}{k}$$

where *δ*exp is the isotope composition expressed as *δ* value of expired air during exercise, *δ*ing is the ^13^C enrichment of ingested CHO (sucrose), and δb is the ^13^C enrichment of expired air during resting (background) before exercise began. The amount of CO_2_ (in litres) produced by the oxidation of 1 g of glucose (0.7467) was k. When water was drunk, the *δ*^13^C values remained similar to those at rest. EXOox remained close to 0 g/min.

Changes in endurance performance were estimated from two points of view. The use of the incremental test data allowed us to calculate the Lactate Threshold (LT) [18,19], the Onset of Blood Accumulation (OBLA) [26], and the peak running velocity reached (V_peak_). Time to fatigue was measured via CRT. It was assumed that the inability to complete a 2 h run due to fatigue would be a clear sign of the expected reduced performance after Everesting. In addition, variations in the measured parameters between the preparation and recovery periods were compared to identify any differences due to Everesting.

### 2.4. Results

**Figure 3:**
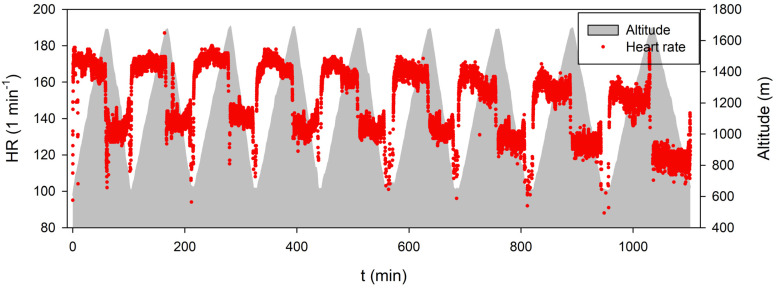
The Everesting event consisted of nine ascents and descents (grey), each beginning and ending at the same location. The HR time course is shown with red dots. Towards the end of Everesting, the time for ascents increased (shown by the more gentle slopes of the grey structures), and the HR increased less significantly.

The subject started the event at 00:00:00 (midnight) and completed the Everesting trial at 18:22:30 (h:min:s) (Figure 3). The distance covered was 81.850 km, and the cumulative terrestrial altitude was 9349 m. The temperature at the start (600 m) was 12 °C. The wind was calm, and fog somewhere on the route. The temperature at the peak of the mountain (1609 m) was 6 °C, and the wind was calm and moderately cloudy. The temperature at the end of the trial was 18 °C, wind calm, moderately cloudy. The average HR during the first four ascent intervals was 170 ± 1 min^−1^ and decreased to 156 ± 4 min^−1^ during the last three ascents (Figure 3). The heart rate was 137 ± 3 min^−1^ during the first six descent intervals and decreased to 125 ± 6 min^−1^ during the last three descent intervals (Figure 3). The runner experienced increased fatigue of his respiratory muscles during the last two ascents. These sensations disappeared during descents.

The subject consumed food with an energy equivalent of about 5830 kcal while Everesting. The runner’s food consisted of energy gels (10 × 400 g), energy bars (6 × 240 g), bananas (6 × 600 g), pasta with tuna (155 g), an isotonic drink (4.5 L), water (2 L) and an energy drink (0.5 L). The consumed drinks and food enabled the runner to maintain body mass within −3% of the pre–event BM.

The runner’s body composition characteristics remained stable during the preparation period (Table 2) but changed during the recovery period after the trial (Table 2). All observed parameters were minimally decreased in a similar interval between 5 and 19 days after the trial. Small changes in the body composition enable comparison between different parameters without corrections.

**Figure 4:**
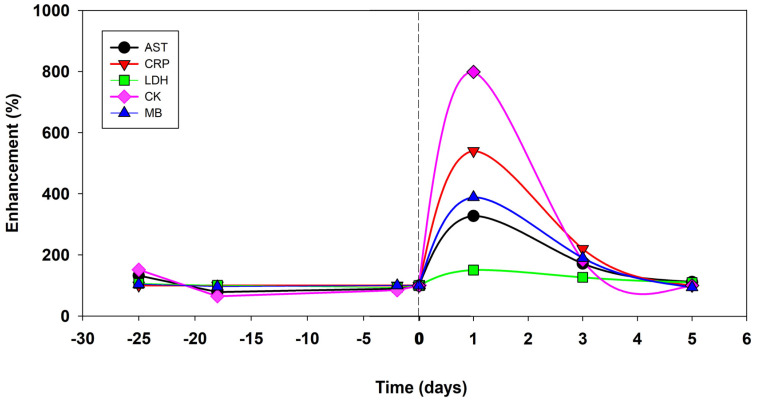
The time course of the fluctuations in blood markers for muscle damage and systemic inflammation shows a synchronous peak on the first day after Everesting and a decrease in this phenomenon on the fifth day after Everesting. The differences between the values during the preparation and recovery phases are obvious.

The haematological resting status was determined using three tests during the preparation period before Everesting and four tests within five days after the trial (Table 3). All markers were stable during the preparatory period (Table 3). During the recovery period, typical changes were seen in the markers for muscle microdamage (CK and Mb), which increased dramatically and peaked on the first day (Table 3, Figure 4).

Markers of systemic inflammation (AST, CRP and LDH) peaked on the first day after the trial (Figure 4). These markers decreased within five days after the trial to the values reached during the preparatory period (Table 3, Figure 4). Decreases in the concentrations of testosterone and cortisol peaked on the first day after the trial but returned to the values measured during the preparatory period after five days (Table 3).

**Figure 5:**
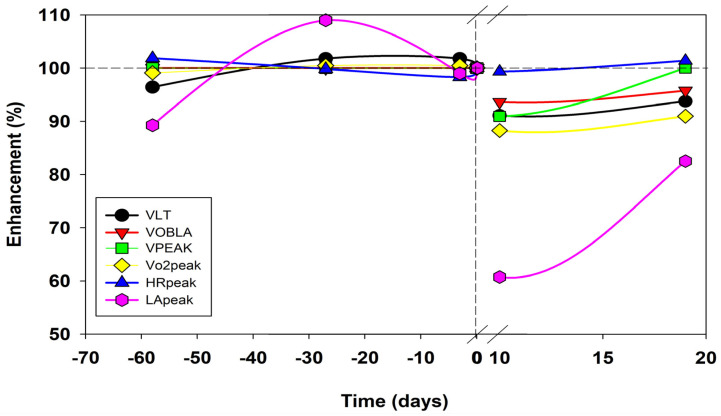
The time course of the relative (%) values of performance characteristics estimated during the incremental test shows a significant decrease in all parameters except HR_peak_ on the 10th day after Everesting. The other variations continued to decrease or remained at constant values. Nevertheless, even on day 20 during the recovery period, these values did not reach the range observed in the preparation period, with the exception of V_peak_.

Performance characteristics typical of submaximal intensity (LT and OBLA) were stable during the preparatory period (Table 4) but remained at 90% of the pre-trial values 19 days after Everesting (Table 4, Figure 5). The peak performance equivalent to running velocity (V_peak_) at maximal intensity during the incremental test (Table 4) was stable at 5.5 m∙s^−1^ during the preparation period but decreased to 5.0 m∙s^−1^ (a 9% decrease) for 5–15 days after the trial (Table 4, Figure 5). On day 19, during the recovery period, the V_peak_ again presented values similar to those in the preparation phase. The CRT tests showed decreased performance due to the shorter running duration. Instead of 120 min, as in the preparation period, the runner stopped running after 60 min due to fatigue. The CRT velocity of 3.9 m∙s^−1^ corresponded to 71% of the V_peak_ during the preparatory period. After Everesting, this value increased to 78% of V_peak_ at 0.5 months. Based on a comparison of Cr within runs during the preparatory period, Cr increased steadily (by 0.02 mL∙kg^−1^∙m^−1^ on average). In each run, Cr reached values that were 0.03 mL∙min^−1^∙m^−1^ (9%) higher than those during the preparatory period after 0.5 months and 0.05 mL∙min^−1^∙m^−1^ (19%) higher than those during the preparatory period about one month after Everesting (Table 5).

Vo_2_ did not change during the incremental test in the preparatory or recovery periods. Peak Vo_2_ values followed the pattern of peak performance (Figure 5) but did not return to pre-trial values. These values remained at 90% after 19 days of recovery (Figure 5). During CRT, Vo_2_ increased about 0.5 months after Everesting and remained elevated one month after this event (Table 5).

**Figure 6:**
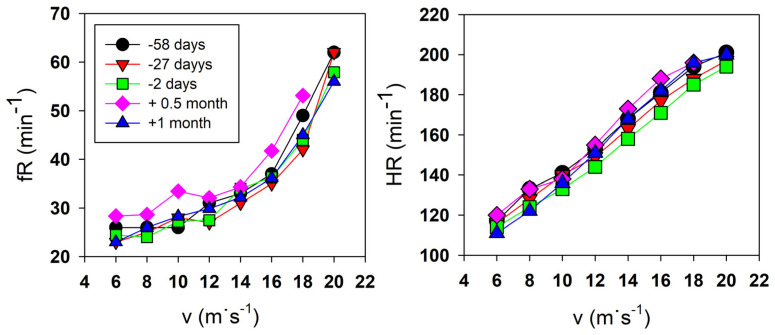
Breathing frequency (fR, **left figure**) and heart rate (HR, **right figure**) during incremental tests repeated throughout the experimental period. The images show relatively stable responses during the preparatory period in March (58 days before Everesting) and April (27 and 2 days before Everesting). The values of fR and HR in May showed higher values about 0.5 months after Everesting, while both fR and HR decreased to values similar to those in the preparatory period about one month after Everesting.

V_E_ did not change after Everesting during the incremental test and remained similar to that in the preparatory period. Nevertheless, fR typically remained elevated until ten days after Everesting and decreased one month after the event to the values measured during the preparatory period (Figure 6). The runner experienced respiratory muscle fatigue and reported chest muscle pain during the CRT on day eight after the trial, so he had to stop the test after 60 min. Increased V_E_ and fR accompanied this phenomenon (Table 5) during the CRT test about 12 days after the trial.

The HR showed a tendency to increase during the incremental test about ten days after Everesting but returned to the values reached during the preparatory period after about one month (Figure 5). HR_peak_ did not change and remained at a similar level (Table 4). During the CRT test, HR increased by 5–8 min^−1^, similar to the results of the tests performed around 0.5 to one month after Everesting (Table 5).

**Figure 7:**
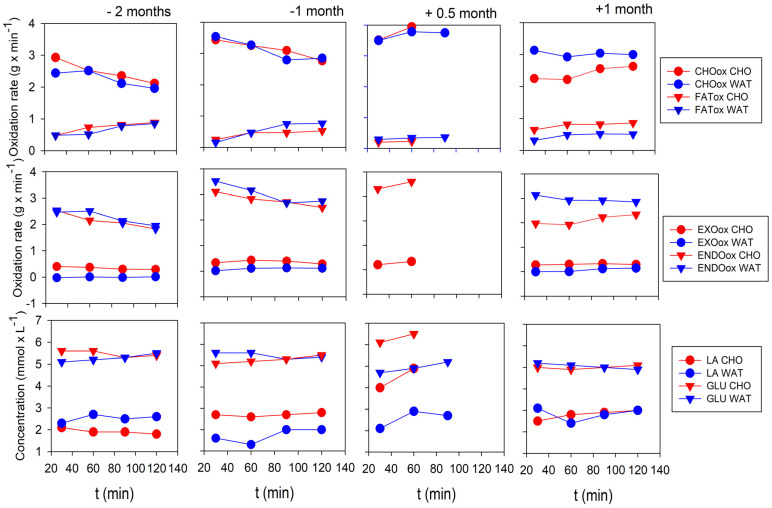
Changes in CHOox and FATox during a two-hour continuous running test at 3.9 m∙s^−1^ (14 km∙h^−1^) (**top row figures**), changes in EXOox (**middle row figures**) and changes in [LA] and [GLU] (**bottom row figures**) when CHO or WAT (water) was consumed throughout the experiment. The figure shows an increase in CHOox over the period of 0.5 months during the recovery period, accompanied by increased [LA] and [GLU], but not EXOox, values.

Carbohydrate and fat metabolism were monitored during CRT. First, the test running velocity of 3.0 m∙s^−1^ presented a 7% higher relative intensity (about 0.5 m∙s^−1^) because V_peak_ and LT decreased during the recovery period, just as they did throughout the preparatory period. Therefore, the presentation of all other results must be interpreted in the context of this phenomenon. CHOox values stabilised or decreased throughout each run and differed little depending on the CHO or WAT beverages consumed during the preparation period (Figure 7, upper figures). The Everesting trial increased CHOox values, which remained unexpectedly high (3.8 g∙min^−1^) about eight days after the Everesting trial (Figure 7, top row of figure). However, the subsequent recovery phase decreased these values, bringing them closer to those of the preparation period (Figure 7, top row of figure). FATox (Figure 7, top row of figure) was lower than CHOox in both the preparatory and recovery periods. FATox achieved its lowest values about eight days after Everesting (Figure 7, top row of figure), which practically reflects a reversal of CHOox changes. EXOox, the oxidation rate from the beverage, presented an expected difference between CHO and WAT (Figure 7, middle row), with no effect of the trial on this parameter. The difference between CHOox and EXOox reflects the oxidation of endogenous carbohydrate sources (ENDOox) (Figure 7, middle row of figure), which showed an unexpectedly large increase to 3.6 g∙min^−1^ 0.5 months after Everesting. About one month after Everesting, this value decreased to 2–2.5 g∙min^−1^ (Figure 7, middle row of figure). The ENDOox time course was associated with [LA] fluctuations during the preparatory and recovery periods (Figure 7, bottom row).

During the incremental test, [LA] showed no difference due to Everesting. The LA_peak_, which dropped to 60% of the pre-trial value around day ten after the Everesting trial (Figure 5), did not return to the pre-trial values. On day 19 of the recovery period, LA_peak_ was 80% (Figure 5). During CRT, [LA] initially fluctuated around 2–3 mmol∙L^−1^ throughout the preparatory period but then increased to 5 mmol∙L^−1^ after about 0.5 months in the recovery period and returned to pre-trial values about one month after Everesting (Figure 7, bottom row of figure).

During CRT, similar to other parameters representing CHO metabolism, [GLU] fluctuated in a steady state at about 5–6 mmol∙L^−1^ during the preparatory period and increased to 6 mmol∙L^−1^ at about 0.5 months during the recovery period (Figure 7, bottom row of figure). Finally, about one month after Everesting, [GLU] decreased to values similar to those in the preparation period (Figure 7, bottom row of figure).

## 3. Discussion

A single runner was observed during a two-month preparatory period for an Everesting mountain ultramarathon and one month during recovery. Decreased running performance during recovery persisted in two phases over one month after the Everesting trial. This confirms the hypothesis of two recovery phases after Everesting. The first phase was characterised by a dramatic decrease in performance that prevented running during the first week after Everesting. This phenomenon was associated with increased blood markers of muscle microdamage and systemic inflammation at rest, which lasted only a few days. This phase disappeared when the blood markers returned to their fluctuation ranges observed during the preparatory period.

Nevertheless, the reduced performance continued into the second phase of the recovery period, with a slow but incomplete return to the values of the preparatory period. This supports the hypothesis that the second recovery phase is extended by a month or longer. A decreased Lactate Threshold, the Onset of Blood Lactate Accumulation, the Vo_2peak_ and peak running velocity determined during the incremental test showed decreased running performance. Consequently, the increased energetic costs of running, heart rate, the increased total carbohydrate oxidation rate (CHOox), endogenous carbohydrate oxidation rate (ENDOox) and blood levels of [LA] and [GLU] were elevated during the 0.5-month recovery period. They typically showed increased relative running intensity at the same absolute running velocity of 14 km∙h^−1^ during constant running tests. Increased ventilation and respiratory rate during the constant running test were typical characteristics of respiratory muscle fatigue and increased relative running intensity. The hypothesis that the characteristics of the second phase can be determined by a continuous running test was confirmed. The changes in the presented parameters throughout the recovery period were partially associated with a return of performance toward the values typical of the preparatory period.

The runner’s performance, as determined by a repeated incremental running test and continuous running test (CRT), did not change during the preparatory period but significantly decreased during the first phase (week) of the recovery period after Everesting. The dramatic increase in blood markers for muscle microdamage (CK and MB) and systemic inflammation (AST and CRP) peaked on the first day after Everesting. It disappeared within one week, in agreement with previous findings [7,10,12,26]. It is expected that depleted or reduced glycogen stores in the legs were also replenished during this phase due to reduced exercise and a normal diet [16,27]. In contrast, the runner’s performance recovered more slowly and became similar to that in the preparation period after about one month during the second phase of the recovery period. The most likely explanation for this difference is an increase in relative running intensity (effort) at a similar absolute running velocity due to decreased performance and slow recovery of the damaged muscle fibres [4,28,29]. This phenomenon was indirectly observed in our study during the incremental test based on a reduced running velocity according to LT and OBLA during the second phase after the first week of the recovery period, with CRT as a shortened time to fatigue. In the subsequent part of the second phase in the recovery period, the values of LT and OBLA remained similarly reduced and showed incomplete recovery. During the one-month recovery period, the time to fatigue for CRT returned to the targeted two hours observed before Everesting. However, fatigue was still present. Notably, a feeling of severe respiratory muscle fatigue in addition to leg fatigue seems to be why the runner stopped CRT at 60 min, 0.5 months after Everesting, and fatigue was still observable one month later in spite of the runner reached the targeted two hours. Decreases in performance after ultramarathons have been reported previously but using different, non-specific, short-term and maximal tests [4,14,15].

The energy cost of running (Cr) in CRT increased during recovery. It was detected after 60 min of running 0.5 months after Everesting and after 120 min of running one month after Everesting. This result primarily reflects increased relative running intensity due to a decrease in V_peak_ by about 0.5 m∙s^−1^ and Vo_2peak_ by 9 mL∙kg^−1^∙min^−1^, both representing decreases of about 10%, compared with the pre–Everesting values, as well as a decrease in submaximal running velocity LT by 0.4 m∙s^−1^ (10%). This result also likely reflects persistent microdamage to the muscle fibres [4,14,15] due to mechanical and oxidative stresses [5,6,9]. The aforementioned changes and microdamage to muscle fibres can be responsible for the high increase in ENDOox and CHOox 0.5 months after Everesting during CRT at running intensities of 80–85% Vo_2max_. As mentioned above, these changes increased relative running intensity and, consequently, influenced metabolic changes. To our knowledge, this is the first mention of this phenomenon during recovery after Everesting.

However, explaining this phenomenon is difficult due to a single case study. Moreover, a typical reference study by Derman et al. [30] shows a similar CHOox of 4 g∙min^−1^ as in ours. The increase in CHOox was not associated with EXOox but may be explained by increased ENDOox, i.e., increased utilisation of glycogen from the limbs and respiratory muscles. The fatigue of the limb muscles was the result of pre-existing microdamage to muscle fibres due to mechanical stress, oxidative stress and glycogen depletion of skeletal muscles during Everesting, whereas the fatigue of respiratory muscles was more likely due to glycogen depletion of the diaphragm [29] and oxidative stress [6]. Although EXOox did not change, the increase in [GLU] may be explained by the greater selection of intramuscular glycogen as fuel. This phenomenon may be due to increased relative running intensity and, consequently, additional recruitment of motor units, resulting in the activation of IIa fibres and/or less well-trained motor units that are less efficient at using fat as fuel [16,30]. Another consequence of the increased activation of muscle fibres was an increased demand for oxygen supply due to increased Cr. This increase in oxygen supply can be achieved by increasing HR, which consistently increases cardiac output and oxygen delivery [16]. Increased fR could be a compensatory response to increased respiratory muscle fatigue [31]. However, whether the respiratory muscles play an important role in this process can only be speculated. On the other hand, both leg and respiratory muscles compete for blood flow when the respiratory muscles are already more fatigued at moderate intensities, especially when the relative running intensity (effort) increases [31,32]. This factor can increase the progression of leg muscle fatigue.

The results of this study should be used with caution due to the limitations of a single case-study design. Generally, lack of rigour, weak possibility for generalization of results, difficult for replication and bias of researcher feeling in interpretations and conclusions are typical sources of limitations. We have tried as much as possible to reduce the effects of the first and fourth limitations by using a large spectre of variables determined in different tests. This research provides novel insights into the recovery period after an extreme endurance run, focusing specifically on Everesting. However, the analysis of the recovery period using three tests and more than twenty characteristics needs to be revised in the future. Instead of testing running performance with the same absolute running velocity, another test should be performed with the same relative running intensity, especially during recovery. However, these changes would further complicate the study design, as the additional testing may strongly interfere with recovery characteristics.

During the CRT tests at 1-month intervals (Figure 6, right column), there was an unexpected increase in CHOox WAT and ENDOox WAT during water consumption. Both values were higher than those of the corresponding CHO drink consumed five days earlier during the previous CRT. A possible explanation for this phenomenon is related to the particular training conditions during this five-day interval. Namely, the first test was performed by consuming CHO. The following five-day training consisted of two long-distance training sessions: a continuous 35 km run of about 3.7 km∙h^−1^ and, three days later, a 25 km Nordic walk in the mountains. Regardless of whether both training sessions were performed for the first time after Everesting, they could influence the renewal of partial muscle microdamage and increase CHOox and ENDOox with the WAT drink.

## 4. Conclusions

Our observations of a single ultramarathon runner during a two-month preparatory period before Everesting and a one-month recovery period after Everesting indicate that a temporary decrease in performance can be detected by tracking various parameters over a relatively long period through repeated observations. Comparing pre-and post-Everesting values allowed us to analyse the extent and time course of post-Everesting enhancements based on calibrated reference values for pre-Everesting fluctuations. This study showed that recovery after Everesting took longer than previously observed from blood markers of muscle damage and systemic inflammation and consisted of two phases. The first phase lasted less than one week, although the decrease in performance was the greatest. The second phase lasted longer than one month and could be recognised by typical changes in various characteristics measured by incremental tests, especially the continuous running test. Candidate characteristics could be employed in constructing a future biological network to describe the recovery period.

## Figures and Tables

**Table 1:** Testing schedule during the experimental period.

Testing Day	−58	−54	−47	−27	−25	−18	−2	0	1	3	5	8	10	12	19	25	30
Incremental test	X			X			X	**TRIAL**					X		X		
Continuous running test		Xw	Xc		Xc	Xw						Xc		Xw		Xc	Xw
Hematological analyses					X	X	X		X	X	X						
Body composition			X			X	X		X	X	X	X			X	X	

X—realised test; Xw—water beverage; Xc—carbohydrate beverage.

**Table 2:** Fluctuations in body composition parameters throughout the preparatory and recovery periods.

	Preparatory Period				Recovery Period					
Testing (Days)	−47	−18	−2	0	+1	+3	+5	+8	+19	+25
Body mass (kg)	69	69	69		69	68	68	67	69	70
BMI (kg∙m^2^)	21.6	21.7	21.7		21.6	21.3	21.3	21	21.2	21.8
Fat mass (kg)	4.6	4.2	4.9		3.5	4.2	4.6	4.7	5.2	5.9
Visceral fat (%)	18.9	17.5	19.2		11.1	11.4	14.1	11.1	14.7	21.8
Muscle mass (kg)	37.3	36.9	37.7		37.7	36.7	36.6	36	36.8	37.4

**Table 3:** Fluctuations in resting blood markers of systemic inflammation and muscle damage throughout the preparatory and recovery periods.

	Preparatory Period				Recovery Period		
Testing (Days)	−25	−18	−2	0	+1	+3	+5
AST (μkat∙L^−1^)	0.86	0.51	0.58		2.13	1.12	0.73
CRP (mg∙L^−1^)	5	5	5		27	11	5
LDH (μkat∙L^−1^)	3.86	3.63	3.37		5.47	4.60	4.00
CK (μkat∙L^−1^)	8.34	3.55	4.64		43.96	10.01	5.55
Mb (μg∙L^−1^)	36	33.3	34.6		134.6	65.9	32.8
Testosterone (μg∙L^−1^)	2.81	2.76	2.84		1.72	1.90	2.77
Cortisol (nmol∙L^−1^)	506	502	519		273	430	490

Abbreviations: AST—aspartate aminotransferase, CRP—C-reactive protein, LDH—lactate dehydrogenase, CK—creatine kinase, Mb—muscle myoglobin.

**Table 4:** Fluctuations of submaximal and maximal performance characteristics are determined via the incremental testing protocol throughout the preparatory and recovery periods.

	Preparatory Period				Recovery Period	
Testing (Days)	−58	−27	−3	0	+10	+19
LT (m∙s^−1^)	3.6	3.8	3.8		3.4	3.5
OBLA (m∙s^−1^)	4.7	4.7	4.7		4.4	4.5
V_peak_ (m∙s^−1^)	5.5	5.5	5.5		5.0	5.5
Vo_2peak_ (ml∙min^−1^∙kg^−1^)	73	74	74		65	67
V_Epeak_ (L∙min^−1^)	160	161	162		137	150
fR_peak_ (min^−1^)	62	62	58		53	56
VT_peak_ (L)	2.58	2.60	2.80		2.58	2.68
HR_peak_ (min^−1^)	201	197	194		196	200
LA_peak_ (mmol·L^−1^)	11.9	14.9	13.2		8.1	11.0

LT—Lactate Threshold; OBLA—Onset of Blood Lactate Accumulation; V_peak_—peak velocity; Vo_2peak_—peak oxygen consumption; V_Epeak_—peak ventilation; fR_peak_—peak respiratory frequency; VT_peak_—peak of tidal volume; HR_peak_—peak heart rate; LA_peak_—peak blood lactate concentration; all values were achieved during the incremental test.

**Table 5:** Responses during the 2 h continuous run at 3.9 m∙s^−1^.

	Preparatory Period								Recovery Period							
	−2 Months				−1 Month				+0.5 Month				+1 Month			
Time (Min)	30	60	90	120	30	60	90	120	30	60	90	120	30	60	90	120
Vo_2_ (L∙min^−1^)	3.422	3.627	3.662	3.634	3.400	3.695	3.588	3.451	3.344	3.712			3.314	3.636	3.893	4.034
Vo_2_ (ml∙min^−1^∙kg^−1^)	50	53	53	53	49	53	54	50	49	55			48	53	56	58
Cr (ml∙min^−1^∙m^−1^)	0.19	0.21	0.21	0.21	0.19	0.21	0.20	0.19	0.19	0.23			0.18	0.22	0.22	0.25
V_E_ (L∙ min^−1^)	80	83	83	82	82	91	87	82	84	95			81	89	96	102
fR (min^−1^)	30	35	37	38	34	40	38	40	35	43			33	40	41	42
HR (min^−1^)	168	174	177	180	163	167	170	175	180	188			170	175	180	185

Vo_2_—absolute and relative oxygen consumption, Cr—energy cost of running; V_E_—ventilation, fR—respiratory frequency, HR—heart rate.

## Data Availability

Due to ethical and privacy issues, the additional data, except those already presented in the manuscript, is not publicly available.
